# The E92K Melanocortin 1 Receptor Mutant Induces cAMP Production and Arrestin Recruitment but Not ERK Activity Indicating Biased Constitutive Signaling

**DOI:** 10.1371/journal.pone.0024644

**Published:** 2011-09-13

**Authors:** Tau Benned-Jensen, Jacek Mokrosinski, Mette M. Rosenkilde

**Affiliations:** Laboratory for Molecular Pharmacology, Department of Neuroscience and Pharmacology, Faculty of Health Sciences, University of Copenhagen, Copenhagen, Denmark; German Institute for Human Nutrition, Germany

## Abstract

**Background:**

The melanocortin 1 receptor (MC1R) constitutes a key regulator of melanism. Consequently, many naturally-occurring MC1R mutations are associated with a change in color. An example is the Glu-to-Lys substitution found at position II:20/2.60 in the top of transmembrane helix II which has been identified in melanic mice and several other species. This mutation induces a pronounced increase in MC1R constitutive activity suggesting a link between constitutive activity and melanism which is corroborated by the attenuation of α-melanocyte stimulating hormone (αMSH) induced activation. However, the mechanism by which the mutation induces constitutive activity is currently not known.

**Methodology/Principal Findings:**

Here we characterize the constitutive activity, cell surface expression and internalization of the mouse mutant, Mc1r E92K. As previously reported, only positively charged residues at position II:20/2.60 induced an increase in constitutive activity as measured by cAMP accumulation and CREB activation. Furthermore, the mutation induced a constitutive recruitment of β-arrestin. This phenomenon is only observed in MC1R, however, as the equivalent mutations in MC2-5R had no effect on receptor signaling. Interestingly, the mutation did not induce constitutive ERK1/2 phosphorylation or increase the internalization rate indicating the constitutive activity to be biased. Finally, to identify regions of importance for the increased constitutive activity of Mc1r E92K, we employed a chimeric approach and identified G102 and L110 in the extracellular loop 1 to be selectively important for the constitutive activity as this, but not αMSH-mediated activation, was abolished upon Ala substitution.

**Conclusions/Significance:**

It is concluded that the E92K mutation induces an active conformation distinct from that induced by αMSH and that the extracellular loop 1 is involved in maintaining this conformational state. In turn, the results suggest that in MC1R, which lacks an extracellular loop 2, the first extracellular loop may play a more prominent role during receptor activation than in general.

## Introduction

**Figure 1:**
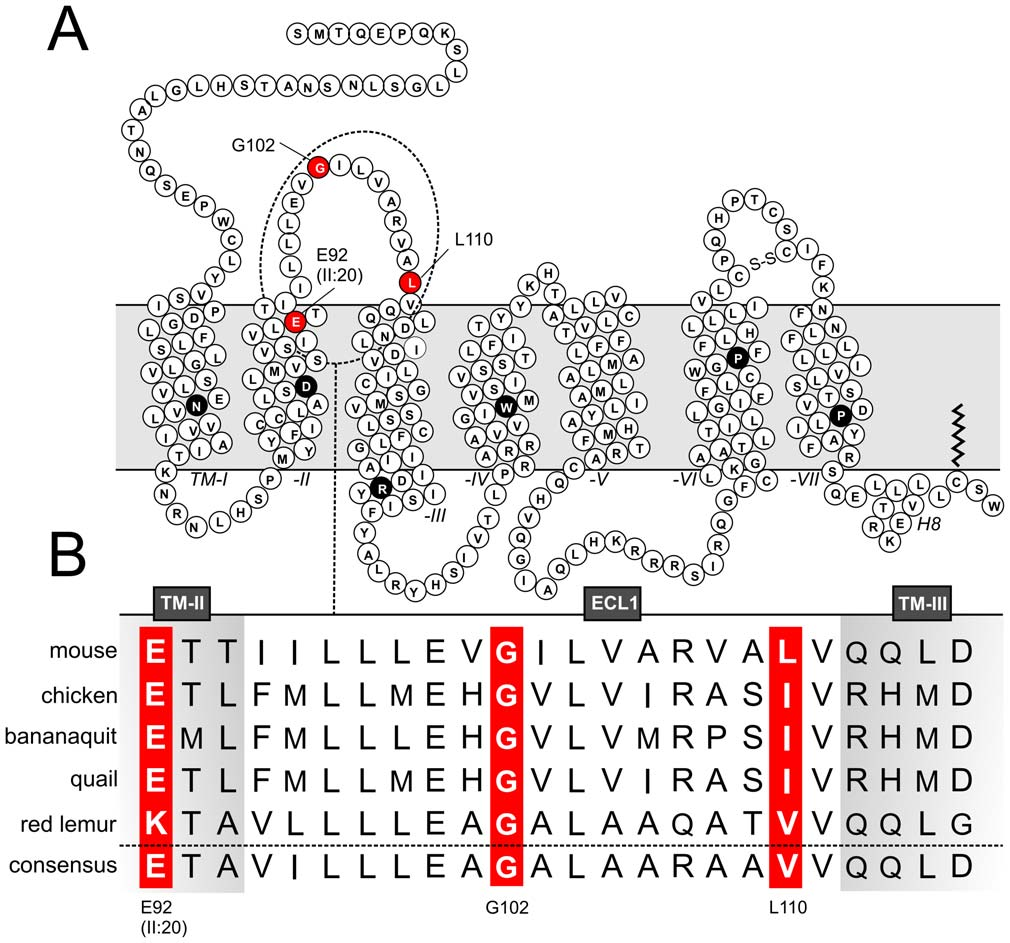
Primary structure of the murine MC1R. **A.** Serpentine model of Mc1r. The highly conserved residues in each transmembrane helix of rhodopsin-like 7TM receptors are colored in black with white letters. The putative disulfide bridge in ECL3 is indicated (-S-S-) as is the palmitoylation site at C313. The key residues in this study, E92 at position II:20/2.60 as well as G102 and L110 in ECL1, are colored in red with white letters. Extracellular and intracellular boundaries of the transmembrane helices were estimated using the TMHMM Server v. 2.0 (www.cbs.dtu.dk/services/TMHMM). **B**. Primary structure of MC1R ECL1. Alignment of the MC1R ECL1s of the five species in which a Glu-to-Lys substitution has been identified at position II:20/2.60 i.e. mouse (*Mus musculus*, accession number NP_032585), chicken (*Gallus gallus* brown leghorn, BAD91484), bananaquit (*Coereba flaveola*, AAK50793), Japanese quail (*Cortunix japonica*, BAD91487) and red lemur (*Varecia rubra*, AAP31013). The consensus ECL1 sequence of MC1R from 83 species is shown at the bottom. The key residues E92, G102 and L110 (mouse numbering) are highlighted in red.

In mammals, melanism is regulated by the levels and distribution of the eumelanin (black/brown) and pheomelanin (red/yellow) pigments. The two major genetic loci involved in the regulation of this process are *extension* and *agouti* encoding the melanocortin 1 receptor (MC1R) and the agouti signaling protein, respectively. MC1R is a constitutively active Gα_s_-coupled seven transmembrane (7TM) receptor which is expressed primarily in epidermal melanocytes and is activated by several proopoimelanocortin derived peptides, most potently by α melanocyte-stimulating hormone (αMSH). However, MC1R is unique among 7TM receptors in that it is also targeted by an endogenously expressed inverse agonist, namely the agouti signaling protein [1]. Accordingly, both constitutive and ligand-induced activity of MC1R can be inhibited by this peptide [2]–[4]. Upon MC1R activation, accumulation of cAMP activates the rate-limiting melanogenic enzyme tyrosinase leading to elevated and reduced levels of eumelanin and pheomelanin, respectively. Thus, ultimately, receptor activation results in a darker phenotype, whereas inhibition of receptor activity results in a lighter phenotype [1]. Structurally, MC1R and the four other melanocortin receptors (MC2-5R) are interesting as they possess three unique features not found in rhodopsin-like 7TM receptors in general. First, they lack the highly conserved cysteine residue in the top of TM-III (CysIII:01, 90% conserved [5]) that normally forms a functionally important disulfide bridge with a cysteine in the extracellular loop (ECL) 2. Absence of this structural hallmark is only found in few other receptors including the cannabinoid and sphingosine receptors. Secondly, the ECL2 of the melanocortin receptors (MCRs) is very short consisting of only 3 polar amino acids keeping TM-IV and -V in close proximity of one another. This loop is on average the longest of the three ECLs [5] and in the currently available 7TM receptor crystal structures ECL2 contains prominent secondary structures such as a β-sheet (rhodopsin [6], [7]), an α-helix (β-adrenergic receptors [8], [9]) or an inter-loop disulfide bridge (adenosine A2_A_ receptor [10]). Finally, the ECL3 of the MCRs is unusually rich in conserved Cys and Pro residues suggesting that it is detained in a specific fold likely by an intra-loop disulfide bridge [11] (Figure 1A).

MC1R as well as MC3-5R have been shown to exhibit a high level of constitutive activity i.e. signaling in the absence of an agonist [2], [11], [12] which likely is physiological important. For instance, in morbidly obese individuals several mutations in MC4R have been identified that selectively abolish constitutive but not ligand-induced MC4R activity [13] and still others affecting both [14]. Given that activation of MC4R has an anorexigenic effect, it is possible that the constitutive activity of MC4R provides a satiety signal. In turn, when absent this could lead to an increase in food intake and ultimately obesity. However, even stronger evidence in favor of constitutive activity being physiologically relevant comes from naturally-occurring mutations in the MC1R. Thus, loss-of-function mutations in the MC1R frequently lead to a lighter phenotype as e.g. seen in individuals with red hair harboring the high penetrance R151C, R160W or D294H mutations [15]. On the other hand, a range of MC1R mutations has been identified in different species that constitutively activate the receptor and are associated with a darker phenotype [16]–[18]. Interestingly, among the latter, one specific mutation is found in several phylogenetically distantly related species, namely the charge-reversing Glu-to-Lys substitution at position II:20/2.60 in the top of TM-II. At the functional level, this mutation induces a high level of constitutive activity and abolishes the activation by αMSH [16], [19]. To date, it has been identified in dominant *extension* alleles associated with a melanic phenotype in the mouse [17], several species of chicken [16], [20]–[22], the Japanese quail (*Coturnix japonica*) [23] and the Bananaquit (*Coereba flaveola*), a passerine bird [24]. Furthermore, it has also been observed in black-and-white ruffed lemurs (*Varecia variegata*) and red ruffed lemurs (*Varecia rubra)* although in these species the mutation is not perfectly linked to a darker coat color [25]. Although several of these species are found in nature the mutation does not seem to have an evolutionary advantage as such [24].

In the present study, we characterize the effect of the mouse Glu-to-Lys MC1R substitution (E92K) on cAMP accumulation, β-arrestin recruitment, cell surface expression, ERK activation and receptor internalization showing that the mutation only affects the two former. Moreover, using a chimeric approach we identify two residues in ECL1 that are crucial for the elevated constitutive activity of the Mc1r E92K mutant thereby suggesting a role for ECL1 in the conformational constraining of MC1R in an active conformation.

## Results

### A positive charge at position II:20/2.60 profoundly increases Mc1r constitutive cAMP accumulation and β-arrestin recruitment but not ERK1/2 phosphorylation

**Figure 2:**
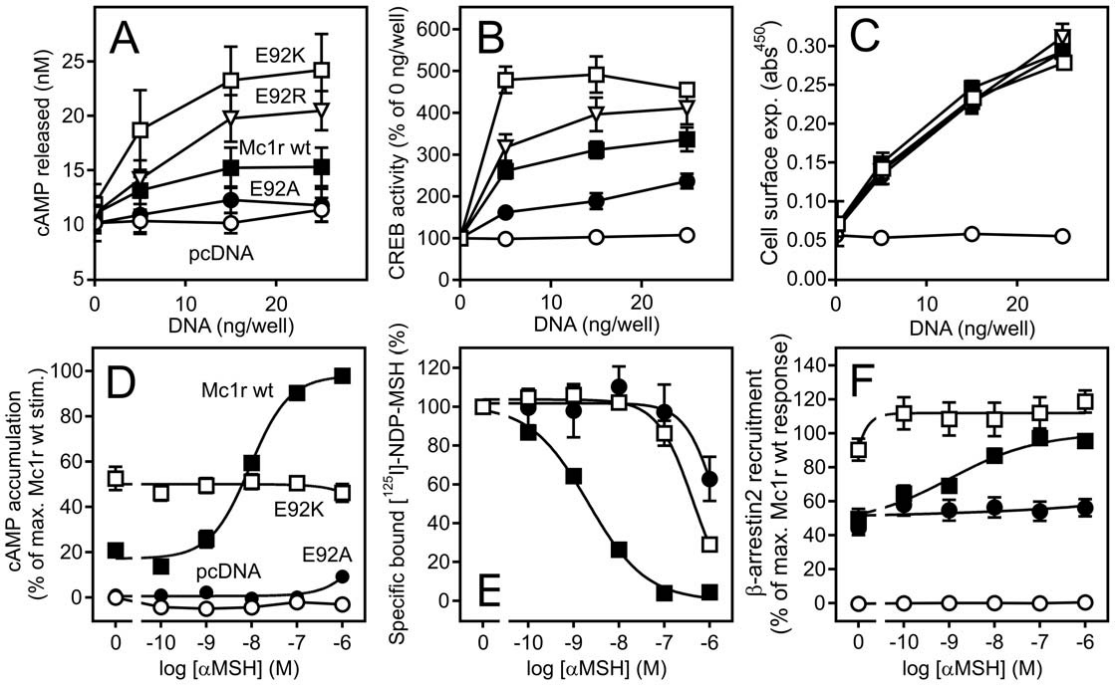
A positive charge at position II:20/2.60 increases the level of MC1R constitutive cAMP accumulation, CREB activity and β-arrestin recruitment. **A**. cAMP accumulation in HEK293 cells transiently transfected with FLAG-tagged Mc1r wt (solid squares), Mc1r E92K (open squares), Mc1r E92R (open triangle), Mc1r E92A (solid circle) or pcDNA (open circles) at 0, 5, 15 or 25 ng DNA per well. The results represent mean ± SEM of raw data from four independent experiments performed in triplicates. **B**. CREB activity reflecting long-term changes in cAMP levels measured in HEK293 cells transiently transfected with CREB-LUC reporter vector and FLAG-tagged Mc1r wt (solid squares), Mc1r E92K (open squares), Mc1r E92R (open triangle), Mc1r E92A (solid circle) or pcDNA (open circles) at 0, 5, 15 or 25 ng DNA per well. The results are normalized to the basal activity (i.e. at 0 ng DNA/well) and represent mean ± SEM of four independent experiments performed in quadruples. **C.** Cell surface expression of the same constructs as in A and B. The results are performed and presented the same way as in A. **D**. αMSH-induced activation as measured by cAMP accumulation n HEK293 cells transiently transfected with FLAG-tagged Mc1r wt (solid squares), Mc1r E92K (open squares), Mc1r E92A (solid circle) or pcDNA (open circles) at 25 ng DNA per well. The results are normalized to background (0%, pcDNA in the absence of αMSH) and the maximal level of agonist-induced stimulation (100%, Mc1r wt at [αMSH]  =  1 µM) and represent mean ± SEM of at least three independent experiments performed in triplicates. **E.** Heterologous competition binding in HEK293 cells transiently transfected with Mc1r wt (solid squares), Mc1r E92K (open squares) or Mc1r E92A (solid circle) at 50 ng DNA/well using [^125^I]-NDP-MSH as radioligand (60 pM). The results have been corrected for non-specific binding (i.e. binding to pcDNA-transfected cells in presence of 1 µM αMSH) and are presented relative to the binding in absence of αMSH in percent as mean ± SEM of three independent experiments. **F.** αMSH-induced β-arrestin recruitment in CHO cells stably expressing a β-arrestin-β-galactosidase fusion construct. The cells were transiently transfected with Mc1r wt-β-gal fusion construct (solid squares), Mc1r E92K construct (open squares), Mc1r E92A construct (solid circle) or vector control (open circles) at 50 ng DNA/well and exposed to increasing concentrations of αMSH. The results are given in percent relative to the maximal αMSH-induced response of Mc1r wt and are mean ± SEM of three independent experiments.

The functional impact of the naturally-occurring E92K mutation (at position II:20/2.60, Figure 1) in the mouse MC1R (Mc1r) was initially examined by measuring cAMP accumulation, CREB activation (reflecting long-term changes in cAMP level) and cell surface expression in transiently transfected HEK293 cells. In agreement with previous findings [17], [19], the E92K mutant displayed a much higher level of constitutive cAMP accumulation and CREB activation compared to the wt receptor (Figure 2A–B). However, this was not due to a difference in cell surface expression (as surface expression and constitutive activity are proportional, [26]) since the expression levels of Mc1r wt and E92K mutant were similar (Figure 2C). It has previously been shown that an increase in constitutive activity only is observed when a positively charged residue (i.e. Lys or Arg) is found at position II:20/2.60 in Mc1r [19]. Supporting this finding, we observed a higher level of constitutive activity for the E92R mutant but a lower for the E92A mutant as compared to the wt receptor although their expression was similar (Figure 2A–C).

Besides the impact of the Mc1r E92 substitutions on constitutive activity, we also examined their effect on αMSH-induced cAMP accumulation in transiently transfected HEK293 cells. As seen in Figure 2D, αMSH dose-dependently activated Mc1r wt with an EC_50_ value of 9.2 ± 1.1 nM. However, as observed before [16], [19], αMSH was not capable of activating neither of the E92 mutants. Compared to the maximal αMSH response, the E92K mutant was approximately 50% constitutively active whereas the E92A mutant did not differ from pcDNA-transfected cells (Figure 2D). To characterize this behavior further, we carried out whole cell heterologous competition binding in transiently transfected HEK293 cells using [^125^I]NDP-MSH as radioligand. The total specific binding of [^125^I]NDP-MSH to the E92K was wt-like whereas it was lower for the E92A mutant. To reach 10% specific binding (of total counts) also in this case, we increased the number of cells. For Mc1r wt, αMSH dose-dependently displaced the radioligand with an IC_50_ of 2.1 ± 0.1 nM (Figure 2E). On the contrary, the affinity of αMSH for the E92A and E92K mutants was much lower showing a >700- and 200-fold decrease in IC_50_ values, respectively. Thus, this demonstrates that the abolished activation of the E92 mutants likely is a result of lack of high-affinity ligand binding. Finally, we characterized the constitutive and αMSH-induced recruitment of β-arrestin by Mc1R wt and the E92 mutants in cells stably expressing a β-arrestin2-β-galactosidase fusion protein. β-arrestin was dose-dependently recruited by αMSH in cells transiently transfected with a Mc1r wt-β-gal fusion constructs with an EC_50_ of 1.0 ± 0.3 nM (Figure 2F) which is in good agreement with the cAMP accumulation EC_50_ value (Figure 2D). Contrary to the wt receptor, αMSH did not recruit β-arrestin in cells transfected with any of the E92 mutants also in agreement with the cAMP accumulation and binding results (Figure 2D-E). However, β-arrestin was also recruited *constitutively* (i.e. in the absence of αMSH). Of note, this recruitment was much more profound for the E92K mutation than Mc1r wt or the E92A mutant thus reflecting the activity profile observed in both the cAMP accumulation and CREB activity assays. Although the activity of the E92A mutant tended to be lower than wt in these assays, no difference in β-arrestin recruitment was observed.

**Figure 3:**
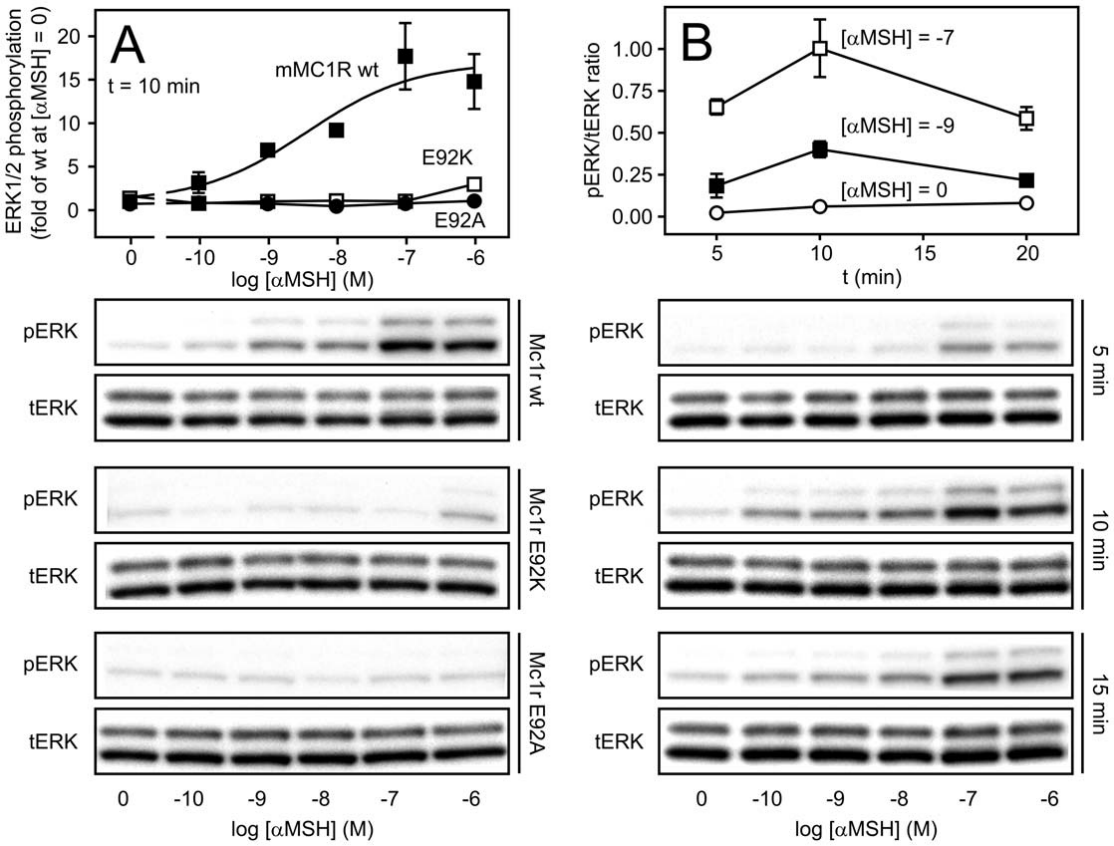
Mc1r-mediated ERK1/2 phosphorylation. **A.** αMSH-induced ERK1/2 phosphorylation in HEK293 cells transiently transfected with Mc1r wt (solid squares), Mc1r E92K (open squares) or Mc1r E92A (solid circles, lower). The data are presented relative to the Mc1r wt receptor-mediated phosphorylation in absence of αMSH indicated as [αMSH] = 0 and are mean ± SEM of three independent experiments. The lower panels show representative blots of phosphorylated ERK1/2 (pERK) and total ERK (tERK) detected on the same membrane. The phosphorylation was measured after 10 min incubation in presence of αMSH. **B.** Kinetic analysis of αMSH-mediated ERK1/2 phosphorylation in HEK293 cells transiently transfected with Mc1r wt. The phosphorylation was measured after 5, 10 and 15 min of incubation. The quantitative data shows the phospho-ERK : total ERK ratio at different αMSH concentrations (indicated as the power of 10) and are mean ± SEM of three independent experiments.

It has previously been demonstrated that agonist-induced activation of the human MC1R leads to phosphorylation and activation of the extracellular signal regulated kinases (ERK) 1 and 2 [27]. To examine whether this also applies to the murine ortholog and E92 mutants, we measured ERK1/2 activation in transiently transfected HEK293 cells. As seen in Figure 3A, αMSH dose-dependently activated ERK1/2 in cells expressing Mc1r wt with an EC_50_ of 3.7 ± 0.5 nM. Furthermore, we also examined the kinetics of the phosphorylation and found this to peak at 10 min after exposure to αMSH (Figure 3B). Contrary to the wt receptor, αMSH was not able to activate ERK1/2 in cells expressing the E92A or the E92K mutants in agreement with the binding experiments although a low response was observed for the E92K mutant at a αMSH concentration of 1 µM. Of note, despite being highly constitutively active with regard to cAMP accumulation (Figure 2), the E92K mutant did not display any constitutive ERK activation indicating biased activation with regard to *constitutive* activity.

### A positive charge at position II:20/2.60 does not constitutively activate MC2-5R

**Figure 4:**
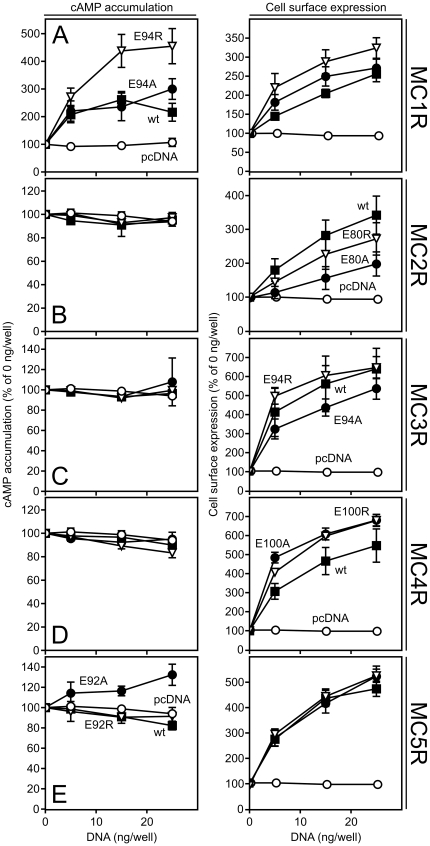
Impact of a positive charge at position II:20 in human MC1-5R. **A–E**. cAMP (left column) and cell surface expression (right column) in HEK293 cells transiently transfected with FLAG-tagged human MC1R (A), MC2R (B), MC3R (C), MC4R (D) or MC5R (E) wt receptor (solid squares), Glu-to-Ala (solid circles), Glu-to-Arg (open triangles) mutants or pcDNA (open circles) at 0, 5, 15 or 25 ng DNA per well. The results are given relative to the background level in the absence of receptor (i.e. at 0 ng/well) as mean ± SEM of at least three independent experiments performed in quadruples.

The five melanocortin receptors (MC1-5R) are up to 45% identical at the protein level. Several residues are conserved among the five members including the Glu at II:20/2.60. To examine whether the same phenomenon applied for the four other melanocortin receptors, we substituted Glu for Arg or Ala in the human MC2-5R and measured the constitutive cAMP accumulation including human MC1R as control. As observed for Mc1r, a Glu-to-Arg substitution at position II:20/2.60 in the human MC1R induced an increase in constitutive activity compared to the wt receptor (1.4-fold at 15 ng DNA/well) whereas the Ala substitution did not (Figure 4A, table 1). Contrary to MC1R, none of the other MCR wt receptors were constitutively active although this has been reported for MC4R previously [28]. However, we did not observe any constitutive activity of this receptor in the CREB assay either or for Mc4r (data not shown). In addition, no increases in activity were observed when an Arg was introduced at II:20/2.60 in MC2-5R (Figure 4B-E, Table 1). For MC5R, the Ala substitution induced an increase in cAMP accumulation (1.2-fold, Figure 4E), however, given this was not observed for the Arg substitution it is likely an unrelated mechanism. In all cases, the wt and mutant constructs were expressed well at the surface (> 2-fold over basal). This was also the case for MC2R although we did not co-express MRAP. Thus, MC1R is the only of the MCRs in which a positive charge at II:20 induces constitutive activity.

**Table 1:** Impact of position II:20/2.60 in the MCR family.

	*Rec./Var.*	*cAMP*			*(n)*	*ELISA*			*(n)*
**MC1R**	wt	261	±	51	3	205	±	11	4
	E94A	236	±	87	3	249	±	25	4
	E94R	437	±	103	3	288	±	31	4
**MC2R**	wt	91	±	9.7	3	282	±	46	4
	E80A	92	±	3.4	3	156	±	34	4
	E80R	93	±	0.6	3	226	±	44	4
**MC3R**	wt	93	±	1.8	3	562	±	146	6
	E94A	93	±	2.2	3	437	±	45	6
	E94R	92	±	2.5	3	605	±	52	6
**MC4R**	wt	97	±	5.3	3	466	±	72	3
	E100A	92	±	5.6	3	608	±	31	3
	E100R	89	±	1.8	3	598	±	20	3
**MC5R**	wt	91	±	3.1	3	437	±	32	5
	E92A	116	±	4.8	3	417	±	38	5
	E92R	91	±	6.0	3	444	±	29	5

Constitutive cAMP accumulation and cell surface expression (ELISA) of the human melanocortin wt receptors (MC1-5R) and II:20/2.60 mutants (Ala and Arg) measured in HEK293 cells transiently transfected with 15 ng DNA/well. The results are given relative to the background level in the absence of receptor (i.e. at 0 ng DNA/well) as mean ± SEM. Rec./Var.: Receptor/Variant.

### Constitutive and ligand-induced internalization of Mc1r wt and E92K mutant

**Figure 5:**
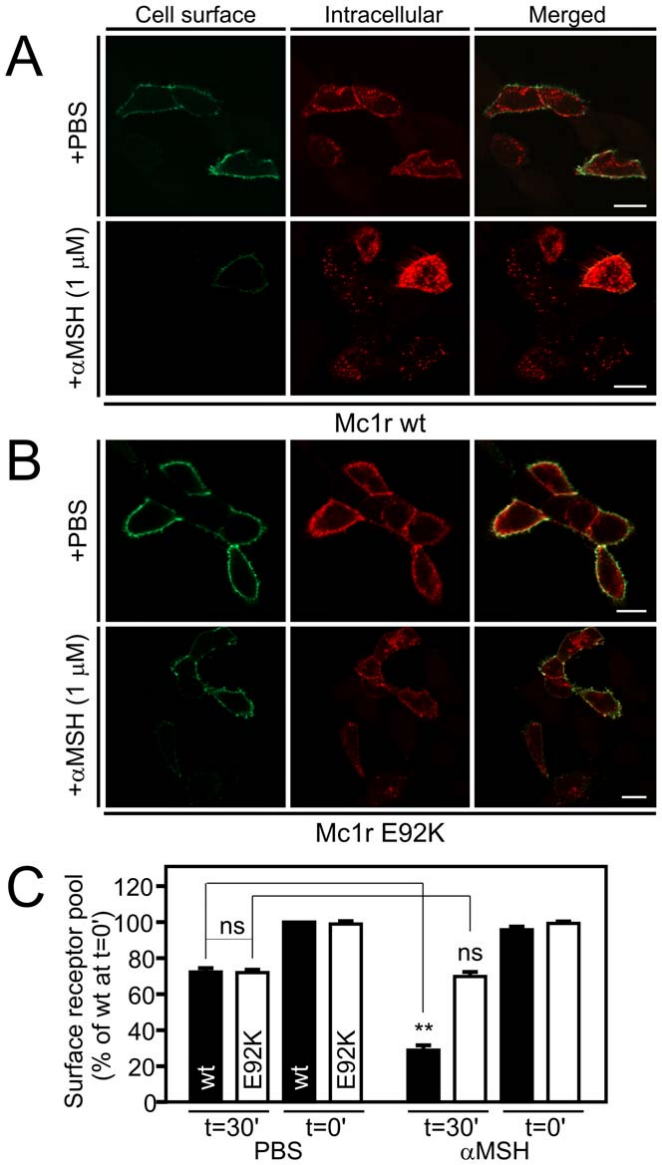
Effect of the E92K mutation on receptor internalization. Antibody-feeding internalization assay. Internalization of FLAG-tagged Mc1r wt (A) or Mc1r E92K (B) in transiently transfected HEK-g cells (150 ng DNA per well) in the presence of vehicle (PBS, upper rows) or αMSH at 1 µM (lower rows). Receptors at the surface were labeled with M1 anti-FLAG antibody prior to an internalization period of 30 min at 37°C. Subsequent to this, labeled receptors still residing at the cell surface were detected before cell permeabilisation (green, left) and internalized receptors after (red, middle) with two different fluorophore-conjugated secondary antibodies (Alexa Fluor 488 and 568, respectively) and analyzed by confocal microscopy. Merged images are presented to the right. Images are representative of two independent experiments. Mock cells were used as controls for unspecific binding of primary or secondary antibodies of which none was seen. Scalebars represent 10 µm. C. ELISA-based internalization assay. Cell surface expression in HEK-g cells transiently transfected with FLAG-tagged Mc1r wt or Mc1r E92K at 25 ng DNA per well either before (t = 0’) or after (t = 30′) an internalization period of 30 min at 37°C in the presence of vehicle (PBS, left) or αMSH at 1 μM (right) as measured by ELISA. The results are given relative to the cell surface expression of Mc1r wt in the absence of ligand (PBS) before internalization (t = 0′) as mean ± SEM of four independent experiments performed in quadruples. ** p<0.01; ns, not significant.

We next examined the effect of the E92K substitution on receptor internalization using two methods. First, we employed an anti-body feeding approach in which cell surface expressed receptors were labeled with primary antibody followed by incubation with either PBS to assess constitutive internalization or αMSH to examine ligand-induced internalization. Subsequently, labeled receptors still residing at the surface and those internalized were separately detected using two different fluorophore-conjugated secondary antibodies before (Figure 5A-B, left panels) and after permeabilisation (middle panels), respectively. Secondly, we also quantified the internalization response using an ELISA-based assay (Figure 5C). As seen Figure 5A and 5B, both Mc1r wt and the E92K mutant were constitutively internalized as labeled receptors were present in endosomes after 30 min incubation with PBS only (red channel, top rows Figure 5A–B). However, a significant part of the labeled receptor pool still resided on the surface (green channel) suggesting the internalization rate to be modest. Accordingly, in both cases only 25% of the receptors were constitutively internalized during incubation as measured by ELISA (Figure 5C). It is interesting to note that despite its high constitutive activity and β-arrestin recruitment, the E92K mutant did not internalize faster than the wt receptor suggesting biased activity like that observed for ERK1/2 phosphorylation. As expected, incubation with αMSH resulted in a prominent internalization of Mc1r wt with only 30% of the receptors still residing at the cell surface after 30 min (Figure 5A bottom row and 5C). On the contrary, αMSH did not induce internalization of Mc1r E92K (Figure 5B bottom row and 5C) which aligns well with the previous notion that αMSH does not activate this mutant. Although recycling of tagged receptors may influence the measurement of internalization, we estimated the extent of recycling within the 30 min incubation period to be 5%. Thus, the contribution of recycling in these assays is minimal.

### ECL1 is involved in the increased level of Mc1r E92K constitutive activity

As an increase in the constitutive activity of MC1R only is observed when a positively charged residue is present at position II:20/2.60 (Figure 2 and [19]), it has previously been speculated that the Lys of Mc1r E92K interacts with a negatively charged residue in the vicinity favoring a more active conformation [19]. However, in the same study, Ala substitution of all negatively charged residues near II:20/2.60 did not abolish the increase in activity suggesting the interaction to be of a different nature. To examine this further we exploited that the corresponding Glu-to-Lys substitution at position II:20/2.60 in Mc4r does not induce constitutive activity (Figure 3). Initially, we analyzed the extracellular subdomains as they are highly divergent between these receptors. To do so we constructed chimeric receptors by swapping the N-terminus, ECL1 and ECL3 of Mc1r with the corresponding Mc4r subdomains. An ECL2 chimera was not generated as this loop consists of only 3 amino acids in the melanocortin receptors (Figure 1). Each swap was done both in wt and E92K background and the cell surface expression and constitutive activity evaluated using ELISA and CREB reporter assays, respectively. We normalized the CREB activity to the expression level (measured at 2.5 ng DNA/well) as these are proportional (Figure S1) to obtain the so-called relative constitutive activity (RCA).

**Figure 6:**
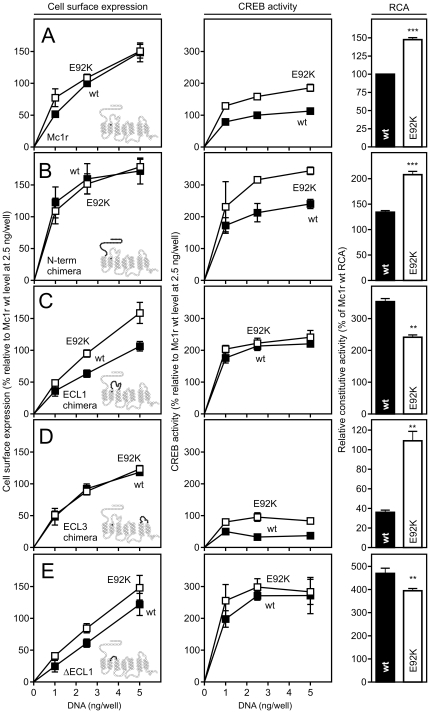
ECL1 is important for the increased constitutive activity of Mc1r E92K. **A–E.** Cell surface expression (left column) and CREB activity (middle column) as measured using ELISA and the CREB reporter assays, respectively, in HEK293 cells transiently transfected with wt (solid squares) or E92K (open squares) FLAG-tagged Mc1r-Mc4r chimeric constructs at 0, 1, 2.5 and 5 ng DNA per well. (A) Mc1r wt and Mc1r E92K, (B) wt and E92K N-terminal chimeras, (C) wt and E92K ECL1 chimeras, (D) wt and E92K ECL3 chimeras and (E) wt and E92K ΔECL1 constructs. In A–D, the serpentine inserts in the left column illustrate which segment of Mc1r (white) has been replaced by the corresponding Mc4r sequence (black). In E, the black segment illustrates the 6 Ala replacing ECL1. The position of E92 in TM-II is also marked in black in all cases. The results has been corrected for background (pcDNA-transfected cells) and are given relative to the Mc1r wt level 2.5 ng DNA/well in percent as mean ± SEM of at least three independent experiments performed in quadruples. The relative constitutive activity (RCA, right column) is calculated for the 2.5 ng DNA per well gene-dose as described in *Materials and methods* and given relative to the RCA of Mc1r wt in percent as mean ± SEM. wt and E92K constructs are depicted in black and white columns, respectively. ** p < 0.01 and *** p < 0.001 by Student's t-test.

All chimeras were expressed well at the surface and their expression levels comparable to that of Mc1r wt (Figure 6A). As expected, the CREB activity of the E92K mutant was much higher than the wt receptor whereas the cell surface expression level was similar. Accordingly, the RCA value of the E92K mutant was higher being increased 1.5 fold (Figure 6A, Table 2). This was also observed for the N-terminus and the ECL3 chimeras where the RCA values of the E92K constructs were 1.5 and 3.0 fold higher than the corresponding wt constructs, respectively (Figure 6B and D, Table 2) indicating that none of these regions are important for the increased constitutive activity induced by the E92K mutation. On the contrary, in the case of ECL1, the RCA value of the E92K construct was 1.5 fold lower (Figure 6C, Table 2). To examine whether this also extends to the human MC1R we generated the equivalent ECL1 constructs in this receptor. Similarly, the RCA value of the corresponding E94K construct was 1.2 lower than the wt construct (Figure S2). To consolidate this finding further, we designed a construct (ΔECL1) in which the ECL1 of Mc1r was replaced by 6 Ala residues. As shown in Figure 6E, both the wt and E92K ΔECL1 constructs were expressed well at the surface although at lower levels than the chimeras. Notably, whereas the cell surface expression of the E92K construct was higher than the wt counterpart, the CREB activity was equivalent to wt resulting in a statistically significant decrease in RCA of 1.4 fold (Figure 6E, Table 2). It should be noted that the domain swapping results in large changes in the RCA values. For the ECL1 and ΔECL1 constructs, the RCA is increased suggesting that ECL1 contains structural elements that stabilize Mc1r in a low activity conformation (Figure 6, Table 2). On the contrary, the RCA of the ECL3 constructs are decreased suggesting this loop to have an opposite function. Collectively, the chimera data indicate that the ECL1 of Mc1r harbors one or more residues essential to the increased constitutive activity of the E92K mutant.

**Table 2:** Importance of ECL1 for the constitutive activation of the E92K mutant.

	*Construct*		*CREB*			*ELISA*			*RCA*			Δ*RCA*	*(n)*
**Mc1r**		wt	100	±	0.0	100	±	0.0	100	±	0.0	1	9
		E92K	159	±	6.9	109	±	5.6	148	±	8.2	1.5	9
**Chimeras**	N-terminal	wt	213	±	29	160	±	24	134	±	6.3		4
		E92K	317	±	7.2	152	±	16	208	±	13	1.5	4
	ECL1	wt	213	±	14	63	±	5.8	353	±	29		9
		E92K	223	±	16	95	±	5.8	241	±	22	0.7	9
	ECL3	wt	33	±	3.1	92	±	7.6	36	±	4		3
		E92K	96	±	13	88	±	5.7	109	±	17	3.0	3
	ΔECL1	wt	271	±	14	61	±	6.5	469	±	51		4
		E92K	299	±	26	84	±	7.2	380	±	21	0.7	4
**Double mutants**	E100N-V101S	wt	96	±	6.9	110	±	7.9	88	±	7.0		4
		E92K	155	±	12	113	±	6.9	139	±	14	1.6	4
	G102T-I103D	wt	127	±	8.9	117	±	7.2	110	±	12		5
		E92K	162	±	9.2	144	±	5.6	113	±	8.3	1.0	5
	L104T-V105D	wt	85	±	7.4	137	±	14	64	±	9.0		3
		E92K	259	±	11	172	±	14	151	±	11	2.4	3
	R107Q-V108S	wt	84	±	7.7	129	±	5.8	65	±	5.5		5
		E92K	158	±	14	150	±	4.3	107	±	13	1.7	5
	A109F-L110T	wt	127	±	24	69	±	6.5	184	±	41		3
		E92K	177	±	11	156	±	11	116	±	12	0.6	3
	G102A-L110A	wt	130	±	16	74	±	16	177	±	12		3
		E92K	116	±	18	97	±	9.3	119	±	15	0.6	3
**Single mutants**	G102T	wt	79	±	3.3	44	±	6.4	190	±	26		4
		E92K	148	±	9.9	131	±	16	116	±	8.3	0.6	4
	G102A	wt	34	±	12	27	±	7.1	124	±	12		3
		E92K	173	±	25	164	±	55	104	±	12	0.8	3
	I103D	wt	94	±	3.6	112	±	7.9	85	±	5.3		4
		E92K	169	±	9.3	158	±	15	109	±	7.3	1.3	4
	L110T	wt	143	±	12	103	±	8.5	141	±	12		4
		E92K	155	±	14	158	±	5.1	99	±	11	0.7	4
	L110A	wt	152	±	50	120	±	16	110	±	24		4
		E92K	353	±	161	200	±	59	163	±	24	1.5	4

Constitutive CREB activity and cell surface expression (ELISA) of wt and E92K Mc1r-Mc4r chimeras and ECL1 mutants in transiently transfected HEK293 cells.

CREB activity and ELISA data are normalized to the Mc1r wt level at 2.5 ng DNA/well and given in percent as mean ± SEM. The relative constitutive activity (RCA) is calculated by normalizing the CREB activity to the cell surface expression at 2.5 ng DNA/well as described in *Materials and methods* and is given relative to the RCA of Mc1r wt. The fold change between the RCA values of the wt and E92K constructs on a given background is presented as ΔRCA.

### ECL1 residues G102 and L110 are essential to the increased level of Mc1r E92K constitutive activity

**Figure 7:**
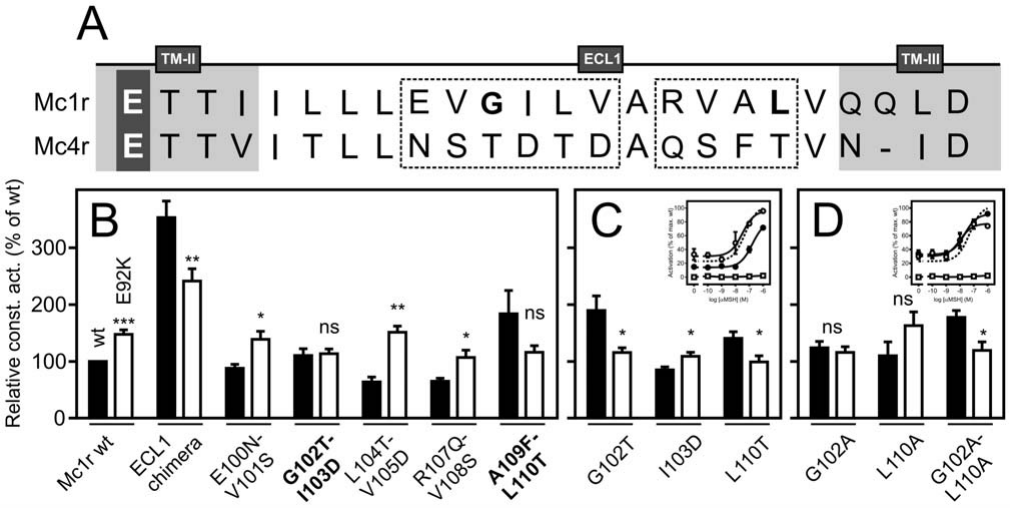
G102 and L110 are essential for the increased constitutive activity of Mc1r E92K but not agonist-induced activation. **A.** Alignment of the Mc1r and Mc4r ECL1 sequences. The stapled box encircles those residues that are not conserved between the two receptors. **B.** RCA values of wt (black columns) and E92K (white) constructs of Mc1r-Mc4r double-swap mutations as well as Mc1r (positive control) and ECL1 chimeras (negative control) at a gene-dose of 2.5 ng DNA per well in transiently transfected HEK293 cells. The results are given relative to the RCA of Mc1r wt in percent as mean ± SEM of at least four independent experiments performed in quadruples. **C.** RCA values of wt and E92K constructs of Mc1r-Mc4r single-swap G102T, I103D and L110T mutations. Performed and presented as in B. *inset*. αMSH-induced activation as measured by CREB activity in HEK293 cells transiently transfected with Mc1r wt (stapled line), G102T (solid circles), L110T (open circles) or pcDNA (open squares) at 25 ng DNA per well. The results are normalized to background (0%, pcDNA in the absence of αMSH) and the maximal level of agonist-induced stimulation (100%, Mc1r wt at [αMSH]  =  1 µM) and represent mean ± SEM of three independent experiments performed in quadruples. **D.** RCA values of wt and E92K constructs of the G102A, L110A and G102A-L110A mutations. Performed and presented as in B. *inset*. αMSH-induced activation as measured by CREB activity in HEK293 cells transiently transfected with Mc1r wt (stapled line), G102A (solid circles), L110A (open circles) or pcDNA (open squares) at 25 ng DNA per well. The results are normalized to background (0%, pcDNA in the absence of αMSH) and the maximal level of agonist-induced stimulation (100%, Mc1r wt at [αMSH]  =  1 µM) and represent mean ± SEM of three independent experiments performed in quadruples. * p < 0.05, ** p < 0.01, *** p < 0.001 and ns, not significant.

To determine whether this is the case, we swapped the ECL1 residues individually. Initially, we examined the ECL1 sequences of Mc1r and Mc4r to determine the degree of conservation. As shown in Figure 7A, only 6 residues (I96, L98, L99, A106, V111 and L114 in Mc1r) are identical or functionally conserved. Initially, we generated 5 double mutations replacing two non-conserved consecutive residues of the Mc1r ECL1 with the corresponding Mc4r residues at a time. All constructs were well expressed at the surface (80–140% of Mc1r wt, Table 2). The E92K constructs of the three mutants E100N-V101S, L104T-V105D and R107Q-V108S exhibited a statistically significant increase in RCA values compared to their wt counterparts thus being functionally similar to Mc1r wt and the E92K mutant (Figure 7B, Table 2). On the contrary, for the G102T-I103D and A109F-L110T mutants we found no statistically significant difference between the RCA values of the wt and E92K constructs (Figure 7B, Table 2). Consequently, the mutants G102T, I103D and L110T were generated to address which residues were important. We did not include the A109 residue as it is not conserved among those species in which a Glu to Lys substitution at position II:20/2.60 has been found (Figure 1B). As seen in Figure 7C, the E92K constructs of the G102T and L110T mutants displayed a significant decrease in RCA values compared to the wt counterparts (1.6 and 1.4 fold, respectively), whereas an increase was observed for I103D (1.3 fold). The decreases observed for these mutants were not merely a result of introduction of Mc4r residues as the wt and E92K RCA values of the corresponding G102A and L110A constructs were similar and even significantly lower (1.5 fold decrease) for the G102A-L110A mutant (Figure 7D, Table 2). Of note, the RCA values of the Mc1r wt and the ECL1 mutants were largely similar and not increased as observed for the ECL1 swap and the ΔECL1 constructs. Thus, collectively, these results indicate that the increase in constitutive activity induced by E92K in Mc1r is specifically dependent on G102 and L110. However, G102 and L110 seem to be important for the constitutive activity only as all mutants were activated with approximately the same potency as Mc1r wt (Figure 7D, inset). Of note, these residues are conserved in all five species in which the Glu-to-Lys substitution has been identified; Gly is fully conserved and the Leu functionally conserved (being Leu, Ile or Val) (Figure 1B) thus further indicating that they are of functional importance.

## Discussion

**Figure 8:**
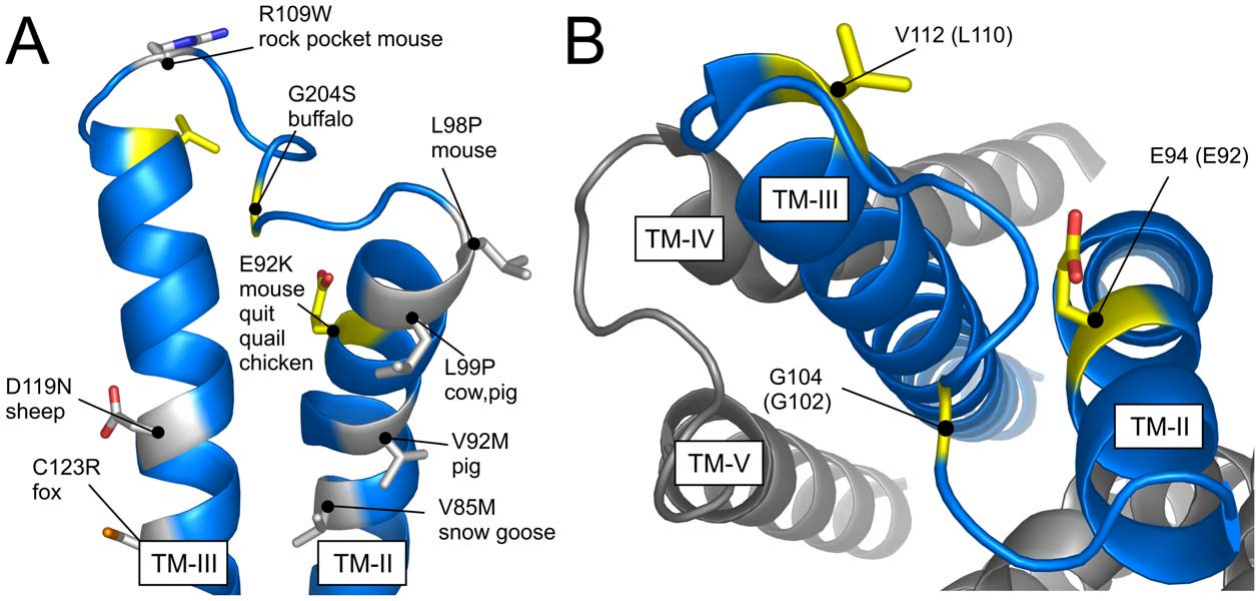
Model of MC1R. **A.** TM-II and -III viewed from within the receptor showing the positions that have been found to be associated with melanism in different species (in grey). The species and type of mutation are presented with the numbering following that of the given species. As in A, the key residues of this study are shown in yellow. **B**. Model of MC1R generated using TASSER [52] as viewed from the extracellular side. TM-II and -III are colored in blue, whereas TM-IV and -V are grey. The equivalent MC1R amino acids to the key residues of this study are colored in yellow being E94 (E92 in the mouse), G104 (G102) and V112 (L110).

The II:20/2.60 Glu-to-Lys substitution in MC1R is highly interesting both from a genetic and a structural perspective. First, it has been identified in the dominant *extension* alleles in five phylogenetically distantly related species rendering it one of the most frequent interspecies mutations in the 7TM receptor superfamily. Second, in all but one (red lemur), the presence of this mutation is associated with a melanic phenotype. Given that the II:20/2.60 mutation has been shown to significantly increase the constitutive activity of both murine (Figure 2 and [17], [19]) and chick [16] MC1R, it is tempting to speculate that the increased level of constitutive activity is the primary cause of melanism in species containing this mutation. This hypothesis is further consolidated by the notion that the ligand-induced activation by the endogenous agonist αMSH is abolished [29]. Also, in several species, constitutively activating mutations have been identified in other MC1R residues which likewise are linked to a darker phenotype. These include the melanic C123R and D121N substitutions found in the Alaska silver fox [18] and sheep or pigs [30], [31], respectively (Figure 8A). Interestingly, these and other activating mutations tend to cluster in the region around TM-II, ECL1 and TM-III in a variety of species and are in general found on the same helix face predominantly facing towards the binding pocket (Figure 8A). Moreover, deletions of TM-II (including II:20/2.60) or ECL1 residues have been identified in the melanic variants of the gray squirrel [32], rabbit [33], Jaguar and Jaguarundis [34]. Although, these MC1R deletions have not been pharmacologically characterized it has been speculated that they either exhibit an elevated level of constitutive activity compared to their wt counterparts or are hyperactive i.e. elicit an amplified agonist-induced response [32]. In favor of the former possibility, the constitutive activity (i.e. RCA) of the Mc1r ΔECL1 construct (which lacks ECL1) was elevated approximately 4.5-fold compared to the Mc1r wt (Figure 6A and E, right panels) but could not be activated by αMSH (data not shown). Of note, we have previously generated similar ΔECL1 constructs in EBI2 and GPR17 (both constitutively active [35], [36]) among others. However, in all cases, removal of ECL1 has been deleterious (unpublished data). In turn, this indicates that the unique absence of the conserved disulfide bridge between TM-III and ECL2 and/or the very short ECL2 of MC1R allows a high degree of structural flexibility in the region around TM-II and -III which putatively could account for the high mutation rate observed in this area.

In the present study, we identified two ECL1 residues, G102 and L110, which are essential for the increased constitutive activity induced by the E92K mutation. Thus, in both cases, the constitutive activity of the E92K construct was either equal to (Ala substitution) or even lower (Thr substitution) than the corresponding wt construct (Figure 7). Importantly, these residues are selectively important for the *constitutive* activity of E92K as the agonist-induced activation by αMSH is not affected by Ala-substitution (Figure 7, insets). Interestingly, a dominant G104S mutation at the equivalent position to G102 has recently been identified in MC1R of the water buffalo [37]. This mutation was perfectly associated with a black coat color and although not pharmacologically characterized it was estimated to be of functional importance by *in silico* analysis. The same G104S mutation has also been found in humans although the functional impact is unclear [38]. Moreover, in the melanic variants of rock pocket mice, one of four MC1R mutations associated with this phenotype is found in ECL1 (a R109W substitution). Finally, in the rabbit, the entire ECL1 has been deleted in one of the pheomelanic MC1R alleles [33]. Thus, collectively, these results suggest that the ECL1 of MC1R indeed is of importance and, in some cases, associated with melanism. This is also indicated by the notion that the ECL1 in MC1R is much longer than that of an average family A 7TM receptor whereas ECL3 is of normal length [5]. In general, ECL2 is the loop of greatest functional importance in the family A [39]. However, given this consists of only 3 amino acids in MC1R, it can be speculated that ECL1 might functionally substitute for ECL2 in this receptor. Moreover, besides MC1R, ECL1 has been shown to be important for ligand binding and receptor activation in several other receptors including the C5a [40], Ste2p [41], neurotensin receptor 1 [42], M4 muscarinic receptor [43] and angiotensin II receptor [44] thus pointing to that not only ECL2 is of functional importance for receptor activation. In addition, several mutations in ECL1 of the MC4R have been linked to obesity including a Thr-to-Met substitution (residue 112) which profoundly decreases the cell surface expression of the receptor [45] and an insertion of an extra adenine resulting in loss of function [46]. Finally, the ECL1 of the thyroid-stimulating hormone receptor (TSHR) is crucial for receptor activation possibly by interacting with the ectodomain [47]. Of note, several ECL1 mutations that alter TSHR constitutive activity have been identified in patients with hypothyroidism (TSHR loss-of-function) [48] or hyperthyroidism (gain-of-function) [49] emphasizing the importance of ECL1 in *in vivo* settings.

It is interesting to note that the E92K mutation only induces constitutive activation of Gαs and β-arrestin recruitment (Figure 2) but does not constitutively activate ERK1/2 (Figure 3) or result in a higher constitutive internalization rate (Figure 5). This is also the case for the constitutive activity of the Mc1R wt and thus indicates that the conformational state of constitutive activity is distinct from that stabilized by the agonist. Alternatively, this difference in activation may be explained by the fact that constitutive conformational states tend to be rather transient whereas agonist-induced states are more stable which could influence interaction with effector proteins [50]. In both scenarios, this could result in differences in e.g. receptor phosphorylation and scaffold protein binding that are important for effector protein activation. Thus, our results collectively indicate that the E92K mutation activates the receptor in a biased mode with regard to the *constitutive* activity. Of note, biased *agonist-induced* activation has recently been reported for MC4R. Thus, as showed by Büch *et al*, MC4R not only couples to Gαs but also Gαi in a hypothalamic cell line [51]. Interestingly, the endogenously expressed Gαs antagonist agouti-related protein was shown to be a Gαi agonist thus activating MC4R in a biased mode.

As an elevated level of MC1R constitutive activity is only achieved when a positively charged residue is present at position II:20/2.60 (Figure 2) this suggests that E92K participates in a functionally important electrostatic interaction. Given the chemical properties of G102 and L110 it is clear that these residues, although important, play an indirect role. Thus, the interaction partner(s) and mechanism of the E92K substitution still remain to be identified. In a previous study, a comprehensive mutational analysis was carried out to address this question [19]. However, although all negatively charged residues in the vicinity of E92K were substituted with Ala, this did not reduce the level of constitutive activity. Interestingly, we have recently reported the same phenomenon in the orphan 7TM receptor EBI2 [26]. Thus, in this receptor an Arg is found at position II:20/2.60 (R87). This residue was shown to be crucial for the constitutive activity as substitution to anything but Lys completely abolished the activity. Thus, analogously to MC1R, a positively charged residue at position II:20/2.60 is required for constitutive activation. However, although we targeted all negatively charged residues in the vicinity of R87, none of these were functionally important. Collectively, these results suggest that in both EBI2 and Mc1r E92K the intramolecular interaction partner(s) of the II:20/2.60 residue, if any, are not negatively charged. One possibility is that the II:20/2.60 residue participates in a cation-pi interaction with one or more unidentified aromatic residues instead. Yet another possibility is that the positively charged II:20/2.60 residue in EBI2 or Mc1r E92K interacts with the negatively charged phosphohead groups of the surrounding lipid bilayer. As seen in the TASSER model of MC1R (Figure 8B) [52] and the current 7TM receptor crystal structures [7]–[10], the residue at position II:20/2.60 is partly facing the lipid environment and partly TM-III depending on the side chain conformation. Thus, given the orientation of II:20/2.60 it is positioned well to interact with phosphohead groups in the vicinity. Although never shown for 7TM receptors, it has recently been demonstrated that a similar interaction exists in voltage-dependent ion channels [53]–[56]. These channels open and close in response to motion of the S4 voltage sensors, which consists of α-helices harboring four or more highly conserved positively charged residues [55], [56]. For instance, by reconstituting purified KvAP, a voltage-dependent K+ channel, into bilayers of different lipid composition it was demonstrated that the presence of lipids containing a negatively charged phosphodiester group was essential for the function of the channel [53]. Future experiments will have to determine whether this also could be the case for 7TM receptors such as EBI2 and the E92K Mc1r mutant. In conclusion, the present study adds to our knowledge about the naturally-occurring Glu-to-Lys substitution at position II:20/2.60 showing that (i) it induces an active conformational state distinct from the agonist-induced and (ii) that this state is highly dependent on ECL1. In turn, these results also substantiate that MC1R indeed possesses unique structural features not found in the majority of 7TM receptor family which likely allow for the high mutation rate observed for this receptor and often results in dramatic phenotype changes.

## Materials and Methods

### Materials

The murine and human MC1Rs (accession numbers NM_008559 and NM_002386) and MC4Rs (NM_016977 and NM_005912) were kindly provided by Birgitte Holst (Laboratory for Molecular Pharmacology, University of Copenhagen, Denmark). Human MC2R (NM_000529), MC3R (AY227893) and MC5R (NM_005913) were purchased from Missouri S&T cDNA Resource Center (www.cDNA.org). Lipofectamine™ 2000 transfection reagent and OPTIMEM were purchased from Life Technologies. SteadyLite (Lyophilized Substrate Solution) was from Packard (Boston, MA). Goat anti-mouse horseradish peroxidase-conjugated antibody was from Pierce (Rockford, IL), while mouse anti-M1-FLAG antibody, forskolin and pertussis toxin were from Sigma Chemicals Co. (St. Louis, MO). Both the SlowFade Antifade reagent, goat anti-mouse Alexa Fluor 488-conjugated and Alexa Flour 568-conjugated antibodies were from Molecular Probes (Carlsbad, CA). The TMB (3,3′,5,5′-tetramethylbenzidine) substrate was purchased from KemEnTech (Taastrup, Denmark). αMSH was from Bachem (Torrance, CA, USA).

### Site-directed mutagenesis

All constructs were inserted into a modified pcDNA3 vector, kindly provided by Kate Hansen (7TM-Pharma, Denmark), which contained an upstream sequence encoding a hemagglutinin signal peptide fused to the M1-FLAG tag. Site-directed mutagenesis was carried out using the *Pfu* polymerase (Stratagene) and the generated mutations were verified by DNA sequencing (MWG Biotech, Martinsried, Germany).

### Transfection and tissue culture

HEK293 cells were grown in DMEM (Invitrogen) adjusted to contain 4500 mg/L glucose, 10% FBS (fetal bovine serum), 180 u/ml penicillin and 45 µg/mL streptomycin (PenStrep) at 10% CO_2_ and 37°C. HEK-g cells (HEK293 cells stably transfected with the macrophage scavenger receptor) were grown in the same medium but with Zeocin (Invitrogen) added at 200 µg/mL. For CREB-luciferase and ELISA assays, transient transfections were carried out using Lipofectamine™ 2000 reagent and the serum-free medium OPTIMEM as described in [26]. Cells were always transfected in parallel for the CREB luciferase and ELISA assays. The CHO-K1 EA-arrestin parental cell line stably expressing β-arrestin fused with β-gal inactive form (DiscoveRx) was cultured in F-12 HAM medium supplemented with 10% FBS, PenStrep and 250 µg/ml Hygromycin B (Invitrogen). Cells were transfected with DNA using FuGENE6 transfection reagent (Roche Diagnostics) according to manufacturer's protocol. For ERK phosphorylation experiments, cells were transfected using the calcium phosphate precipitation method.

### cAMP assay

Levels of cAMP were measured using the HitHunter cAMP XS+ kit according to the manufacturer's recommendations (DiscoveRx, Fremont, CA, USA). HEK293 cells were seeded at 20,000 cells/well in 96-well plates and transiently transfected the following day with FLAG-tagged receptor constructs or pcDNA at the indicated concentrations. Twenty-four hours later, cells were washed twice with HBS buffer (20 mM HEPES, 150 mM NaCl, pH 7.4) and incubated 30 min at 37°C in HBS containing 1 mM IBMX phosphodiesterase inhibitor (Sigma). If used, ligands were then added followed by a 25 min incubation at 37°C. Subsequently, antibody reagent and lysis solutions were added and the plates incubated 1 h on a shaker. Finally, enzyme solution was added and the plates incubated by over-night incubation on a shaker. The luminescence was subsequently measured using a TopCounter (Packard).

### CREB trans-reporter luciferase assay

The trans-reporter CREB-luciferase assay was used to measure long-term changes in intracellular cAMP levels. HEK293 cells were seeded at 35,000 cells/well in poly-L-lysine-coated 96-well plates and transiently transfected the following day with FLAG-tagged receptor constructs or pcDNA at the indicated concentrations along with the trans-activator plasmid pFA2-CREB and the reporter plasmid pFRLUC at 6 ng/well and 50 ng/well, respectively. If used, ligands were added 5 h prior to assay start. The CREB activity was determined 24 h after transfection using the SteadyLite substrate (Perkin Elmer). Briefly, cells were washed twice in Dulbecco's PBS (0.9 mM CaCl_2_, 2.7 mM KCl, 1.5 mM KH_2_PO_4_, 0.5 mM MgCl_2_, 137 mM NaCl, and 8.1 mM Na_2_HPO_4_) and the luminescence measured 10 min after addition of the substrate using a TopCounter. Every receptor construct was tested at least thrice in quadruples.

### Enzyme-Linked Immunosorbent Assay (ELISA)

HEK293 cells were transiently transfected with the indicated FLAG-tagged receptor constructs as described above. Twenty-four hours after transfection, the cells were fixed in 4% formaldehyde for 10 min, washed twice in TBS and blocked for 30 min with TBS containing 2% BSA. Subsequently, the cells were incubated with mouse anti-FLAG M1 antibody at 2 µg/ml for 2 h in TBS supplemented with 1% BSA and 1 mM CaCl_2_. After three washes in TBS containing 1 mM CaCl_2_, the cells were incubated 1 h with goat anti-mouse horseradish peroxidase-conjugated antibody diluted 1∶1000. Following wash, the immune reactivity was determined by addition of TMB substrate according to manufacturer's instruction. All steps were carried out at room temperature.

### Relative constitutive activity (RCA)

The relative constitutive activity was determined at a gene-dose of 2.5 ng/well and calculated according to the equations:
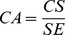
(1)

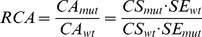
(2)where CA denotes constitutive activity, CS denotes CREB signaling, SE denotes surface expression (measured by ELISA) and RCA denotes relative constitutive activity. wt denotes wild type and mut denotes mutant.

### ERK1/2 phosphorylation assay

HEK293 cells were seeded out in 12-well plates and transfected using the calcium phosphate method with FLAG-tagged Mc1r wt, Mc1r E92K or pcDNA (1.1 µg/well). The cells were serum starved overnight and incubated with PBS or αMSH at 1 µM for 10 min. Subsequently, the cells were washed twice, lysed in lysis buffer (100 mM Tris, 4% SDS, 20% glycerol and spun down for 5 min at 1500 rpm and 4°C. The protein concentration was determined using a BCA protein kit (Pierce). 15-20 µg protein was loaded on Bis-Tris 10% NuPAGE gels and run for 1.5 h at 140 V followed by blotting onto a ethanol-activated PVDF membrane for 1.5 h at 30 V. The membrane was then blocked in TBST (1X TBS with 0.1% Tween20) containing 5% BSA followed by incubation with rabbit anti-phopho ERK IgG antibody (1∶1000). The membrane was subsequently incubated in blocking buffer containing goat anti-rabbit-IgG HRP-conjugated antibody (1∶10000) and developed using SuperSignal West Pico substrate (Pierce). The amount of phosphorylation was measured using a FlourChem H2A camera. The membrane was subsequently stripped using Pierce stripping buffer (Pierce) and the procedure was then repeated with rabbit anti-ERK IgG antibody to detect total ERK.

### Antibody-feeding internalization assay

For the antibody-feeding assays, HEK-g cells (which stably express the macrophage scavenger receptor) were used as they adhere better to the surface and are not lost during washing. HEK-g were seeded on coverslips in 6-well plates at 5⋅10^5^ pr well. The following day the cells were transfected with FLAG-tagged Mc1r wt or Mc1r E92K at 150 ng/well using Lipofectamine™ 2000. Twenty-four hours after transfection, the cells were incubated in ice-cold DMEM medium containing mouse M1 anti-FLAG antibody at 2 µg/mL and incubated 1 h at 4°C. After three washes in cold DMEM medium, the specimens were either immediately fixed in 4% paraformaldehyde or incubated in pre-warmed DMEM medium containing either vehicle (PBS) or αMSH (1 µM) at 37°C for 30 min to induce internalization and then fixed. Subsequently, the coverslips were blocked with TBS containing 2% BSA. To specifically detect labelled receptors still residing at the cell surface, the cells were incubated with goat anti-mouse Alexa Flour 488-conjugated antibody diluted 1∶1000 in TBS containing 1% BSA for 30 min. After washing, the cells were permeabilized and blocked using TBS containing 1% BSA and 0.2% saponin for 30 min. To detect internalized labelled receptors, the coverslips were then incubated with goat anti-mouse Alexa-Fluor 568-conjugated antibodies diluted 1∶1000 in TBS containing 1% BSA for 30 min. Subsequent to washing, the specimens were mounted in SlowFade Antifade reagent using nail polish as sealing. All steps following the internalization step were carried out at room temperature. Mock transfected cells were included to ensure no unspecific binding of any of the antibodies. For all images the laser power and channel gains were kept constant and the slides blinded. For each slide, 10 randomly chosen cells were examined.

### Confocal microscopy

Confocal microscopy was performed using a LSM 510 laser scanning unit coupled to an inverted microscope with a 63x 1.4 numerical aperture oil immersion Plan-Apochromat objective (Carl Zeiss). Alexa-Fluor 488 and 586 were excited using an argon-krypton laser (λ  =  488 nm) and a He-Ne laser (λ  =  543 nm), respectively. Images were recorded in 1024×1024 pixels and averaged over 16 whole frame scans.

### ELISA internalization assay

HEK-g cells were seeded at 35,000 cells/well in 96-well plates and transiently transfected the following day with FLAG-tagged Mc1r wt, Mc1r E92K or pcDNA at 25 ng/well using Lipofectamine™ 2000. Twenty-four hours after transfection, the cells were incubated in cold DMEM containing mouse anti-FLAG M1 antibody at 2 µg/ml for 1 h at 4°C. After three washed in cold DMEM, the cells were either fixed immediately in 4% formaldehyde (t = 0′) or incubated at 37°C in pre-warmed DMEM containing either vehicle (PBS) or αMSH (1 µM) for 30 min (t = 30′) to induce internalization and then fixed. Subsequently, the cells were blocked for 30 min with PBS containing 2% BSA and then incubated 1 h with goat anti-mouse horseradish peroxidase-conjugated antibody diluted 1∶1000 in PBS containing 1% BSA. Following three washes, the immune reactivity was determined by addition of TMB substrate according to manufacturer's instruction. All steps after incubation with primary antibody were carried out at room temperature. The extent of recycling was estimated by stripping receptors still residing on the cell surface after 30 min of incubation and measuring the emergence of tagged receptors within an hour. After 30 min of incubation approximately 5% of the tagged receptor pool had been recycled.

### β-arrestin recruitment assay

Ligand-induced and constitutive recruitment of β-arrestin by Mc1R was studied using the PathHunter β-Arrestin Assay (DiscoveRx) which utilizes an enzyme fragment complementation technology. cDNA for the Mc1r wt and mutants (E92A, E92K) was C-terminally fused with a ProLink tag (DiscoveRx) encoding accessory linker and the small fragment of β-galactosidase (β-gal) and cloned into the pcDNA3.1(+) vector. Assays were performed in a CHO-K1 EA-arrestin parental cell line (DiscoveRx) stably expressing β-arrestin fused with β-gal inactive fragment. Cells were transfected using FuGENE6 transfection reagent according to manufacturer's protocol. Briefly, one day prior to transfection, cells were seeded out in white 96-well plates at a density of 20 000 cells/well. Culture medium was exchanged with OptiMEM I reduced serum medium (50 µL/well). DNA:transfection-reagent complexes were prepared by diluting 0.15 µl FuGENE6 reagent and 50 ng DNA in 50 µl OptiMEM I reduced serum medium (all values given for a single well) and added to cells after 15 min incubation at ambient temperature. Cells were incubated overnight followed by replacement of the OptiMEM I medium with assay medium (culture medium without antibiotics). The β-arrestin recruitment assay was performed 48 hours after transfection. Cells were stimulated with range of concentrations of α-MSH for 90 min. Upon β-arrestin binding to ProLink-tagged Mc1r, complementation occurs between the β-gal fragments forming an active enzyme. Thus, β-arrestin recruitment to the receptors was detected as β-gal activity using the PathHunter detection kit (DiscoveRx). Luminescence signal was measured 60 min after addition of chemiluminescent substrate using a TopCounter.

### Competition binding assay

HEK293 cells were seeded out in 24-well plates at a concentration that obtained 10% specific binding of the added radioligand, [^125^I]-NDP-MSH (Perkin Elmer). For Mc1r wt and the E92K mutant, cells were seeded out at 15,000 cells/well whereas 50,000 cells/well were used for the E92A mutant. The cells were transfected using Lipofectamine2000 and 50 ng DNA/well. One day after transfection, cells were assayed by competition binding for 3 h at 4°C using varying concentrations of unlabelled α-MSH and 60–70 pM [^125^I]-NDP-MSH in 50 mm Hepes buffer pH 7.4, supplemented with 1 mm CaCl_2_, 5 mm MgCl_2_, and 0.5% (w/v) bovine serum albumin. After incubation, cells were washed two times in 4 °C binding buffer supplemented with 0.5 m NaCl. After addition of lysis buffer, the amount of binding was measured using a γ-radiation counter (Wallac). Nonspecific binding was determined as the binding in the presence of 1 µM unlabeled α-MSH. Determinations were made in duplicates.

## Supporting Information

Figure S1 **Gene-dosing of Mc1r wt and Mc1r E92K.** **A–B**. CREB activity (A) and cell surface expression (B) in HEK293 cells transiently transfected with 11 gene-doses ranging from 0 to 5 ng DNA per well of Mc1r wt (solid squares) or Mc1r E92K (open squares) and CREB-LUC reporter vector. The results are presented as mean ± SEM of background-corrected (pcDNA-transfected cells) data of three independent experiments performed in quadruples. The stapled line indicates the 2.5 ng DNA per well gene-dose chosen for RCA value calculation. **C**. Correlation of data from A and B normalized to the CREB activity and cell surface expression values of Mc1r wt at 5 ng DNA per well in percent.
(TIF)

Figure S2 **ECL1 chimera of human MC1R.** **A**. CREB activity (left panel) and cell surface expression (middle panel) in HEK293 cells transiently transfected with human MC1R wt (solid squares) or human MC1R E94K (open squares). The results are given relative to the value at 0 ng DNA per well as mean ± SEM of background-corrected (pcDNA-transfected cells) data of two independent experiments performed in quadruples. The RCA value is given to the right and is presented relative to the RCA of human MC1R wt in percent as mean ± SEM. **B**. CREB activity (left panel) and cell surface expression (middle panel) in HEK293 cells transiently transfected with mMC4R-Mc1r ECL1 wt (solid squares) or E94K (open squares) chimeras and CREB-LUC reporter vector. The results as well as the RCA value (right) are presented as in A. The serpentine insert in A indicate the position of E94 (black) whereas it in B indicates the hMC4R ECL1 (black) substituted into human MC1R (white). * p<0.05.
(TIF)

## References

[1] Garcia-Borron JC, Sanchez-Laorden BL, Jimenez-Cervantes C (2005). Melanocortin-1 receptor structure and functional regulation.. Pigment Cell Res.

[2] Eberle AN, Bodi J, Orosz G, Suli-Vargha H, Jaggin V (2001). Antagonist and agonist activities of the mouse agouti protein fragment (91-131) at the melanocortin-1 receptor.. J Recept Signal Transduct Res.

[3] Lu D, Willard D, Patel IR, Kadwell S, Overton L (1994). Agouti protein is an antagonist of the melanocyte-stimulating-hormone receptor.. Nature.

[4] Siegrist W, Drozdz R, Cotti R, Willard DH, Wilkison WO (1997). Interactions of alpha-melanotropin and agouti on B16 melanoma cells: evidence for inverse agonism of agouti.. J Recept Signal Transduct Res.

[5] Mirzadegan T, Benko G, Filipek S, Palczewski K (2003). Sequence analyses of G-protein-coupled receptors: similarities to rhodopsin.. Biochemistry.

[6] Park JH, Scheerer P, Hofmann KP, Choe HW, Ernst OP (2008). Crystal structure of the ligand-free G-protein-coupled receptor opsin.. Nature.

[7] Palczewski K, Kumasaka T, Hori T, Behnke CA, Motoshima H (2000). Crystal structure of rhodopsin: A G protein-coupled receptor.. Science.

[8] Warne T, Serrano-Vega MJ, Baker JG, Moukhametzianov R, Edwards PC (2008). Structure of a beta1-adrenergic G-protein-coupled receptor.. Nature.

[9] Cherezov V, Rosenbaum DM, Hanson MA, Rasmussen SG, Thian FS (2007). High-resolution crystal structure of an engineered human beta2-adrenergic G protein-coupled receptor.. Science.

[10] Jaakola VP, Griffith MT, Hanson MA, Cherezov V, Chien EY (2008). The 2.6 angstrom crystal structure of a human A2A adenosine receptor bound to an antagonist.. Science.

[11] Holst B, Schwartz TW (2003). Molecular mechanism of agonism and inverse agonism in the melanocortin receptors: Zn(2+) as a structural and functional probe.. Ann N Y Acad Sci.

[12] Sanchez-Mas J, Hahmann C, Gerritsen I, Garcia-Borron JC, Jimenez-Cervantes C (2004). Agonist-independent, high constitutive activity of the human melanocortin 1 receptor.. Pigment Cell Res.

[13] Srinivasan S, Lubrano-Berthelier C, Govaerts C, Picard F, Santiago P (2004). Constitutive activity of the melanocortin-4 receptor is maintained by its N-terminal domain and plays a role in energy homeostasis in humans.. J Clin Invest.

[14] Tarnow P, Schoneberg T, Krude H, Gruters A, Biebermann H (2003). Mutationally induced disulfide bond formation within the third extracellular loop causes melanocortin 4 receptor inactivation in patients with obesity.. J Biol Chem.

[15] Duffy DL, Box NF, Chen W, Palmer JS, Montgomery GW (2004). Interactive effects of MC1R and OCA2 on melanoma risk phenotypes.. Hum Mol Genet.

[16] Ling MK, Lagerstrom MC, Fredriksson R, Okimoto R, Mundy NI (2003). Association of feather colour with constitutively active melanocortin 1 receptors in chicken.. Eur J Biochem.

[17] Robbins LS, Nadeau JH, Johnson KR, Kelly MA, Roselli-Rehfuss L (1993). Pigmentation phenotypes of variant extension locus alleles result from point mutations that alter MSH receptor function.. Cell.

[18] Vage DI, Lu D, Klungland H, Lien S, Adalsteinsson S (1997). A non-epistatic interaction of agouti and extension in the fox, Vulpes vulpes.. Nat Genet.

[19] Lu D, Vage DI, Cone RD (1998). A ligand-mimetic model for constitutive activation of the melanocortin-1 receptor.. Mol Endocrinol.

[20] Kerje S, Lind J, Schutz K, Jensen P, Andersson L (2003). Melanocortin 1-receptor (MC1R) mutations are associated with plumage colour in chicken.. Anim Genet.

[21] Takeuchi S, Suzuki H, Yabuuchi M, Takahashi S (1996). A possible involvement of melanocortin 1-receptor in regulating feather color pigmentation in the chicken.. Biochim Biophys Acta.

[22] Takeuchi S, Suzuki S, Hirose S, Yabuuchi M, Sato C (1996). Molecular cloning and sequence analysis of the chick melanocortin 1-receptor gene.. Biochim Biophys Acta.

[23] Nadeau NJ, Minvielle F, Mundy NI (2006). Association of a Glu92Lys substitution in MC1R with extended brown in Japanese quail (Coturnix japonica).. Anim Genet.

[24] Theron E, Hawkins K, Bermingham E, Ricklefs RE, Mundy NI (2001). The molecular basis of an avian plumage polymorphism in the wild: a melanocortin-1-receptor point mutation is perfectly associated with the melanic plumage morph of the bananaquit, Coereba flaveola.. Curr Biol.

[25] Haitina T, Ringholm A, Kelly J, Mundy NI, Schioth HB (2007). High diversity in functional properties of melanocortin 1 receptor (MC1R) in divergent primate species is more strongly associated with phylogeny than coat color.. Mol Biol Evol.

[26] Benned-Jensen T, Rosenkilde MM (2008). Structural motifs of importance for the constitutive activity of the orphan 7TM receptor EBI2: analysis of receptor activation in the absence of an agonist.. Mol Pharmacol.

[27] Herraiz C, Journe F, Abdel-Malek Z, Ghanem G, Jimenez-Cervantes C (2011). Signaling from the Human Melanocortin 1 Receptor to ERK1 and ERK2 Mitogen-Activated Protein Kinases Involves Transactivation of cKIT.. Mol Endocrinol.

[28] Srinivasan S, Lubrano-Berthelier C, Govaerts C, Picard F, Santiago P (2004). Constitutive activity of the melanocortin-4 receptor is maintained by its N-terminal domain and plays a role in energy homeostasis in humans.. J Clin Invest.

[29] Sanchez MJ, Olivares SC, Ghanem G, Haycock J, Lozano Teruel JA (2002). Loss-of-function variants of the human melanocortin-1 receptor gene in melanoma cells define structural determinants of receptor function.. Eur J Biochem.

[30] Kijas JMH, Wales R, Tornsten A, Chardon P, Moller M (1998). Melanocortin Receptor 1 (MC1R) Mutations and Coat Color in Pigs.. Genetics.

[31] Vage DI, Klungland H, Lu D, Cone RD (1999). Molecular and pharmacological characterization of dominant black coat color in sheep.. Mamm Genome.

[32] McRobie H, Thomas A, Kelly J (2009). The genetic basis of melanism in the gray squirrel (Sciurus carolinensis).. J Hered.

[33] Fontanesi L, Tazzoli M, Beretti F, Russo V (2006). Mutations in the melanocortin 1 receptor (MC1R) gene are associated with coat colours in the domestic rabbit (Oryctolagus cuniculus).. Anim Genet.

[34] Eizirik E, Yuhki N, Johnson WE, Menotti-Raymond M, Hannah SS (2003). Molecular genetics and evolution of melanism in the cat family.. Curr Biol.

[35] Rosenkilde MM, Benned-Jensen T, Andersen H, Holst PJ, Kledal TN (2006). Molecular pharmacological phenotyping of EBI2. An orphan seven-transmembrane receptor with constitutive activity.. J Biol Chem.

[36] Benned-Jensen T, Rosenkilde MM (2010). Distinct expression and ligand-binding profiles of two constitutively active GPR17 splice variants.. Br J Pharmacol.

[37] Miao Y, Wu G, Wang L, Li D, Tang S (2010). The role of MC1R gene in buffalo coat color.. Sci China Life Sci.

[38] Bastiaens MT, ter Huurne JA, Kielich C, Gruis NA, Westendorp RG (2001). Melanocortin-1 receptor gene variants determine the risk of nonmelanoma skin cancer independently of fair skin and red hair.. Am J Hum Genet.

[39] Peeters MC, van Westen GJ, Li Q, Ijzerman AP (2011). Importance of the extracellular loops in G protein-coupled receptors for ligand recognition and receptor activation.. Trends Pharmacol Sci.

[40] Klco JM, Nikiforovich GV, Baranski TJ (2006). Genetic Analysis of the First and Third Extracellular Loops of the C5a Receptor Reveals an Essential WXFG Motif in the First Loop.. Journal of Biological Chemistry.

[41] Hauser M, Kauffman S, Lee BK, Naider F, Becker JM (2007). The First Extracellular Loop of the Saccharomyces cerevisiae G Protein-coupled Receptor Ste2p Undergoes a Conformational Change upon Ligand Binding.. Journal of Biological Chemistry.

[42] Harterich S, Koschatzky S, Einsiedel J, Gmeiner P (2008). Novel insights into GPCR-peptide interactions: mutations in extracellular loop 1, ligand backbone methylations and molecular modeling of neurotensin receptor 1.. Bioorg Med Chem.

[43] Nawaratne V, Leach K, Felder CC, Sexton PM, Christopoulos A (2010). Structural determinants of allosteric agonism and modulation at the M4 muscarinic acetylcholine receptor: identification of ligand-specific and global activation mechanisms.. J Biol Chem.

[44] Fillion D, Lemieux G, Basambombo LL, Lavigne P, Guillemette G (2010). The amino-terminus of angiotensin II contacts several ectodomains of the angiotensin II receptor AT1.. J Med Chem.

[45] Nijenhuis WAJ, Garner KM, van Rozen RJ, Adan RAH (2003). Poor Cell Surface Expression of Human Melanocortin-4 Receptor Mutations Associated with Obesity.. Journal of Biological Chemistry.

[46] Farooqi IS, Keogh JM, Yeo GSH, Lank EJ, Cheetham T (2003). Clinical Spectrum of Obesity and Mutations in the Melanocortin 4 Receptor Gene.. New England Journal of Medicine.

[47] Jaeschke H, Neumann S, Kleinau G, Mueller S, Claus M (2006). An aromatic environment in the vicinity of serine 281 is a structural requirement for thyrotropin receptor function.. Endocrinology.

[48] Sura-Trueba S, Aumas C, Carre A, Durif S, Leger J (2009). An inactivating mutation within the first extracellular loop of the thyrotropin receptor impedes normal posttranslational maturation of the extracellular domain.. Endocrinology.

[49] Biebermann H, Winkler F, Handke D, Gruters A, Krude H (2011). Molecular description of non-autoimmune hyperthyroidism at a neonate caused by a new thyrotropin receptor germline mutation.. Thyroid Res.

[50] Kobilka BK, Deupi X (2007). Conformational complexity of G-protein-coupled receptors.. Trends Pharmacol Sci.

[51] Büch TRH, Heling D, Damm E, Gudermann T, Breit A (2009). Pertussis Toxin-sensitive Signaling of Melanocortin-4 Receptors in Hypothalamic GT1-7 Cells Defines Agouti-related Protein as a Biased Agonist.. Journal of Biological Chemistry.

[52] Zhang Y, Devries ME, Skolnick J (2006). Structure modeling of all identified G protein-coupled receptors in the human genome.. PLoS Comput Biol.

[53] Schmidt D, Jiang QX, MacKinnon R (2006). Phospholipids and the origin of cationic gating charges in voltage sensors.. Nature.

[54] Xu Y, Ramu Y, Lu Z (2008). Removal of phospho-head groups of membrane lipids immobilizes voltage sensors of K+ channels.. Nature.

[55] Jiang Y, Ruta V, Chen J, Lee A, MacKinnon R (2003). The principle of gating charge movement in a voltage-dependent K+ channel.. Nature.

[56] Jiang Y, Lee A, Chen J, Ruta V, Cadene M (2003). X-ray structure of a voltage-dependent K+ channel.. Nature.

