# Supplementary figures and images for: The E92K Melanocortin 1 Receptor Mutant Induces cAMP Production and Arrestin Recruitment but Not ERK Activity Indicating Biased Constitutive Signaling
Source: PLoS One. 2011 Sep 13;6(9):e24644. doi: 10.1371/journal.pone.0024644 (PMC3172247; doi:10.1371/journal.pone.0024644)

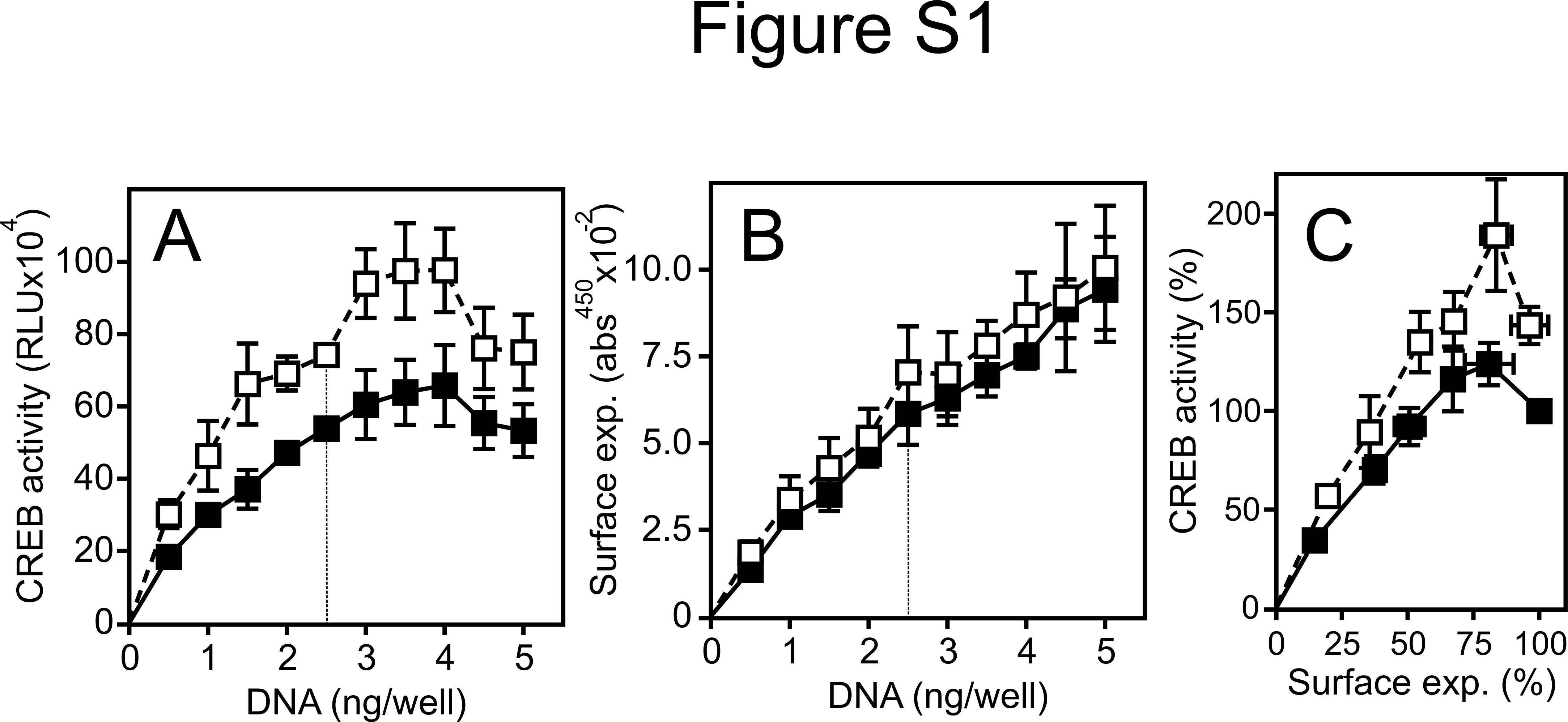

Supplement: Figure S1 — Gene-dosing of Mc1r wt and Mc1r E92K. A–B. CREB activity (A) and cell surface expression (B) in HEK293 cells transiently transfected with 11 gene-doses ranging from 0 to 5 ng DNA per well of Mc1r wt (solid squares) or Mc1r E92K (open squares) and CREB-LUC reporter vector. The results are presented as mean ± SEM of background-corrected (pcDNA-transfected cells) data of three independent experiments performed in quadruples. The stapled line indicates the 2.5 ng DNA per well gene-dose chosen for RCA value calculation. C. Correlation of data from A and B normalized to the CREB activity and cell surface expression values of Mc1r wt at 5 ng DNA per well in percent. (TIF) [file pone.0024644.s001.tif]

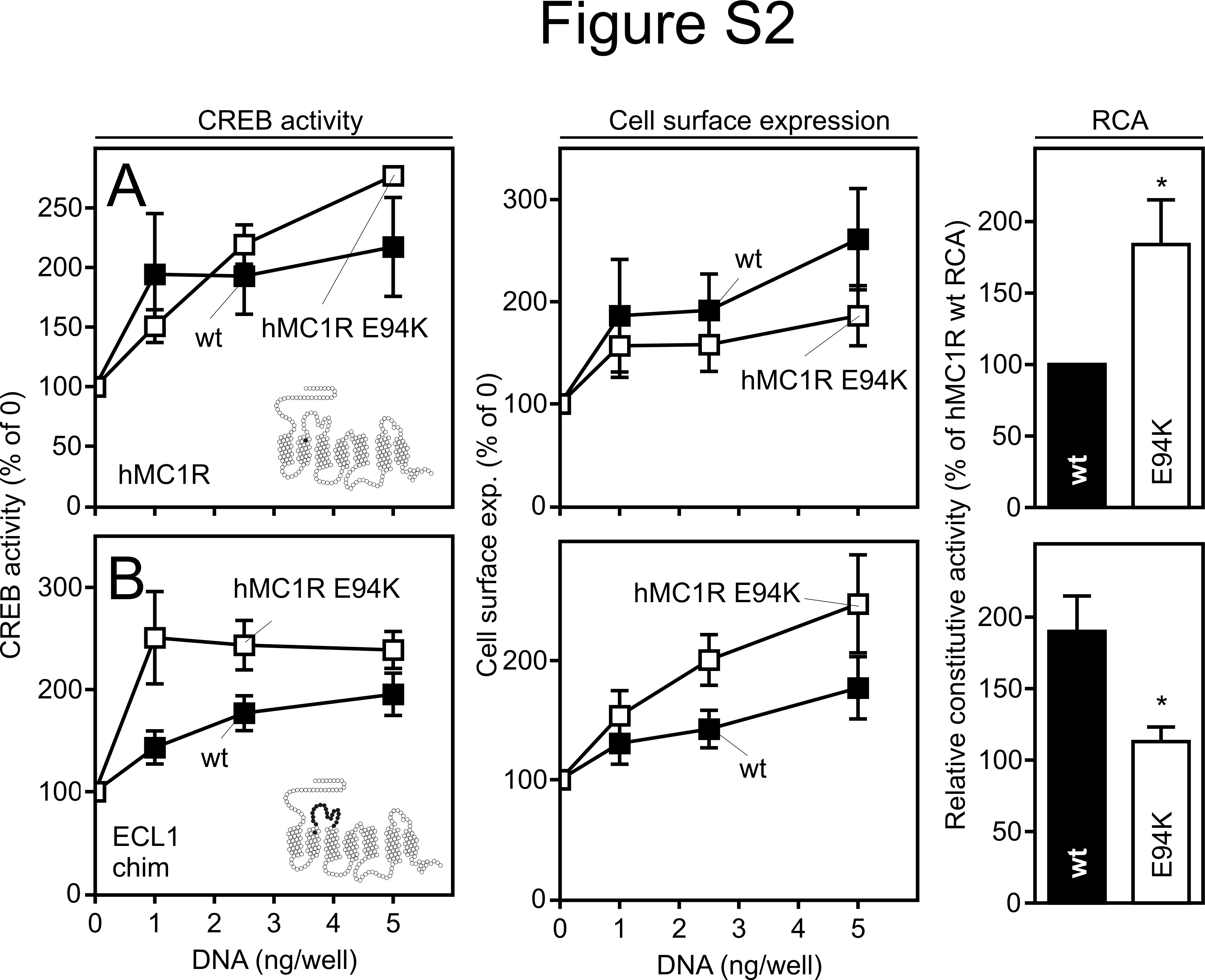

Supplement: Figure S2 — ECL1 chimera of human MC1R. A. CREB activity (left panel) and cell surface expression (middle panel) in HEK293 cells transiently transfected with human MC1R wt (solid squares) or human MC1R E94K (open squares). The results are given relative to the value at 0 ng DNA per well as mean ± SEM of background-corrected (pcDNA-transfected cells) data of two independent experiments performed in quadruples. The RCA value is given to the right and is presented relative to the RCA of human MC1R wt in percent as mean ± SEM. B. CREB activity (left panel) and cell surface expression (middle panel) in HEK293 cells transiently transfected with mMC4R-Mc1r ECL1 wt (solid squares) or E94K (open squares) chimeras and CREB-LUC reporter vector. The results as well as the RCA value (right) are presented as in A. The serpentine insert in A indicate the position of E94 (black) whereas it in B indicates the hMC4R ECL1 (black) substituted into human MC1R (white). * p<0.05. (TIF) [file pone.0024644.s002.tif]
